# Ethnobotany in the Nepal Himalaya

**DOI:** 10.1186/1746-4269-4-24

**Published:** 2008-12-02

**Authors:** Ripu M Kunwar, Rainer W Bussmann

**Affiliations:** 1 Ethnobotanical Society of Nepal, G.P.O. Box 19225, Kathmandu, Nepal; 2 William L. Brown Center, Missouri Botanical Garden, St. Louis, MO, USA

## Abstract

**Background:**

Indigenous knowledge has become recognized worldwide not only because of its intrinsic value but also because it has a potential instrumental value to science and conservation. In Nepal, the indigenous knowledge of useful and medicinal plants has roots in the remote past.

**Methods:**

The present study reviews the indigenous knowledge and use of plant resources of the Nepal Himalayas along the altitudinal and longitudinal gradient. A total of 264 studies focusing on ethnobotany, ethnomedicine and diversity of medicinal and aromatic plants, carried out between 1979 and 2006 were consulted for the present analysis. In order to cross check and verify the data, seven districts of west Nepal were visited in four field campaigns.

**Results:**

In contrast to an average of 21–28% ethnobotanically/ethnomedicinally important plants reported for Nepal, the present study found that up to about 55% of the flora of the study region had medicinal value. This indicates a vast amount of undocumented knowledge about important plant species that needs to be explored and documented. The richness of medicinal plants decreased with increasing altitude but the percentage of plants used as medicine steadily increased with increasing altitude. This was due to preferences given to herbal remedies in high altitude areas and a combination of having no alternative choices, poverty and trust in the effectiveness of folklore herbal remedies.

**Conclusion:**

Indigenous knowledge systems are culturally valued and scientifically important. Strengthening the wise use and conservation of indigenous knowledge of useful plants may benefit and improve the living standard of poor people.

## Background

The term ethnobotany was coined by John W. Harsberger in 1896 [1] and was considered as the art of collection of useful plants by a group of people and the description of the uses of plants. Ford developed the science of ethnobotanical study [2] and included the understanding of knowledge systems through the use of anthropological methods [3]. Over the last century, ethnobotany has evolved into a scientific discipline that focuses on the people-plant relationship in a multidisciplinary manner, incorporating not only collection and documentation of indigenous uses but also ecology, economy, pharmacology, public health, and other disciplines [4]. Presently, ethnobotany has become increasingly valuable in the development of health care and conservation programs in different parts of the world [5]. Ethnobotanical studies that explore and help to preserve knowledge are therefore urgently needed before traditional folklores are lost ever [6].

Ethnomedicine, a branch of ethnobotany, is a set of empirical local practices embedded in the indigenous knowledge of a social group often transmitted orally from generation to generation [7] with intent to understand social, cultural, and economic factors [8] influencing health problems and to overcome such problems. It is a suitable source of information regarding useful medicinal plants that can be targeted for sustainable domestication and management [9]. Ethnobotany and ethnomedicine as sciences addressing indigenous knowledge and practices therefore are now very important for establishing management programs [10].

### Justification

The dialectical relationship between indigenous knowledge and practices shape, the ecosystem and affects the constituent plant populations [11]. By incorporating indigenous knowledge and use in the process of scientific research, new hypotheses for the sustainable conservation of the resources can be developed [12]. Indigenous knowledge and use have to be analyzed to develop appropriate management measures that build on both scientific and local knowledge [13]. Due to changing perception of the local people, and the ever increasing influence of global commercialization and socio-economic transformation, indigenous knowledge on plant resource use is constantly diminishing [14,15]. Due to the lack of organized sustainable and scientifically monitored cultivation and harvesting, proper management techniques, and lack of awareness of social factors, the number of useful plant resources is decreasing at an alarming rate [16]. Furthermore, the indigenous knowledge on the use of lesser-known plants is also rapidly declining [17]. The present study therefore reviews the indigenous knowledge and use of plant resources of Nepal Himalayas along the altitudinal and longitudinal gradient, an area so far unstudied in Nepal.

### Context

The first scientific study of Nepalese useful and medicinal plants was conducted by Francis Buchanan, who collected plants from 1802–1803. He was followed by Nathaniel Wallich 1820–1821 [18]. Don [19] and Wallich [20] also collected plants in the Nepal Himalayas and recorded their uses. The earliest published ethnomedicinal-botanical study of Nepal was a paper on medicinal and food plants from eastern Nepal by Banerji [21]. His study was followed by many other researches [e.g. [22-30]]. Since the 1980s, extensive ethnobotanical studies were conducted. However, none of them has analyzed patterns of the usage of plant resources along the altitudinal and longitudinal gradient. All previous studies enumerated only the plant diversity and usage of plant resources of selected small sites and selected tribal groups, without attempting to synthesize the plant use information in Nepal.

### Review and field visits

A total of 264 studies, with emphasis on ethnobotany, ethnomedicine, and diversity of medicinal and aromatic plants, conducted between 1979 to 2006, were consulted for the present analysis. Of these papers only 76 were including data on the diversity and distribution of medicinal and aromatic plants, ethnobotany, as well as aspects of ethnomedicine and conservation. Four field visits were carried out in May and December 2006, January–February 2007 and April–May 2008, focusing on the Baglung, Baitadi, Dadeldhura, Darchula, Doti, Gulmi, and Kanchanpur districts of west Nepal, stretching from 80°5' to 83°15' E longitude 28°27' 30" to 30°15' N latitude and 390 m to 4570 m altitude. Field visits were meant for data verification and cross checking.

Nepal extends along the Himalayan range between the latitudes 26°22' – 30°27' and longitudes 80°04' – 88°12'. Altitudes vary from less than 60 m in the lowland of Terai in the South to the crest of the Himalaya reaching 8848 m in the North. In the present study, for easier interpretation and analysis along the longitudinal gradient, the country's longitude was divided into three bio-geographical regions: West Nepal (80°E to 83°E), Central Nepal (83°E to 86°E) and East Nepal (86°E to 88°E), [31,32]. An analysis of the altitudinal gradient was made in 1000 m intervals as 60 – 1000 m, 1000 – 2000 m, 2000 – 3000 m, and 3000 – 4000 m.

## Results and discussion

### History and plant usage

Plants have been one of the most important sources of food and medicine since the dawn of human civilization. Archaeological evidence of 60,000 year-old Neanderthal burial grounds in Shanidar, Iraq, points to the use of plants like Marshmallow, Yarrow and Groundsel, which are still used in contemporary folk medicine [33]. Evidence for the medicinal use of *Papaver somniferum*, the opium poppy, dates back 8000 years [34,35]. Concomitantly, the earliest written record of plants used as medicine originating from the Himalayas are found in the 6,500 year old texts of the Rigveda [36], followed by Atharveveda (2000-1000 BC) and Auryveda (600-100 BC) [37,38]. The oldest account recording the uses of 278 Nepalese medicinal plants is 'Saushrut Nighantu,' written in 878 AD (935 BS). Later 'Nepali Nighantu,' an elaborated encyclopedia with information on the traditional knowledge of 750 plant species was published by the Royal Nepal Academy in 1969 [39].

Until the middle of the 19^th ^century, plants were the main therapeutic agents used by humans, and even today almost 80% of the world population rely to some extent on medicinal plants for their primary healthcare needs. The use of nearly 3000 plant species as food during the course of human civilization has been documented, but only about 150 species have been cultivated [40] and less that 10 plant species are meeting over 90% of the world food demand [41]. Human survival still can not be imagined without plants. The importance of plants is substantial and reflected in the large variety of products such as food, fiber, fodder, vegetables, medicinal plants, and aromatic plants. The contribution of medicinal plants in Nepal is important [42]. A total of 1012 useful plants are documented for Nepal [43], of which 554 (54.74%) have ethnomedicinal in properties. An review of research conducted in the Nepal Himalayas indicated that nearly 40% of the studies were related to medicinal plants and ethnomedicine [43], underlining that ethnomedicine is utmost important in the Nepal Himalayas.

### Medicinal plants and their distribution

About 60% of the world population and 60–90% of the population of developing countries (80% in Nepal [44], 70% in India, 80% in Pakistan, 65% in Sri Lanka, 90% in Bangladesh, 85% in Burma, and 60% in Indonesia [45] rely on traditional medicine [46], and about 85% of the traditional remedies for primary health care are derived from plants [47].

In the world, 10–18% of all plant species are used medicinally [48] while in Nepal medicinal plants account about 20–28% of the local flora [49-54]. The data for India and China is significantly higher i.e. 44% and 29–41% respectively [55,56] (Table 1). The present analysis found that an average of 56% of higher plants were ethnobotanically important, and 54% were used as ethnomedicine in the Nepal Himalayas (Table 2). This indicates that there are more ethnobotanically and ethnomedicinally important plant species in the Nepal Himalayas than previously estimated. Similar accounts of a much higher incidence of plants used in ethnomedicine (more than the estimated 10–18% [57] was recorded all over the world; e.g. about 24% in Costa Rica [58], 25–42% in Kenya [59], 27% in Israel [60], 31–51% in Brazil [61], and 46% in Guyana [62], 64% in Jammu Kashmir, India [63], 54% in Similipahar, Orissa, India [64], and 48% in Beni, Bolivia [65].

**Table 1:** Ethnobotanical and ethnomedicinal plants in the world

**SN**	**Location and total no. of higher plants**	**Ethnobotanical plants as % of total flora**	**Ethnomedicinal plants**	**Source**
1	Nepal, 7000	28%	21% of total flowering plants	[49,51]
2	Nepal, 7000	56.35% *	53.59% of total plants in use	Present study
3	Hindu Kush Himalaya, 25000	33–40%		[120]
4	Indian Himalaya, 8000		21% of total flowering plants	[55]
5	India, 17000	14%	44% of total flowering plants	[122]
6	Pakistan, 6000		12% of total flowering plants	[103]
7	China, 29700		41% of total flowering plants	[121]
8	World, 297000 <		10–18% of total plants	[57]

* Percentage of plants used in terms of species richness of sites

**Table 2:** Useful plants of Nepal Himalaya

**Ecological zones {m}**	**Distribution of medicinal plants {%}**			**Average {%}**	**% of ethnobotanically important plants**			**Average {%}**	**% of ethnomedicinally important plants**			**Average {%}**
	W	C	E		W	C	E		W	C	E	
60–1000	32.06	44.28	42.85	39.73 49.20 [36]	40.72 (3)	-	68.20 (1)	54.46	56.76 (8)	38.55 (7)	51.57 (6)	48.96
1000–2000	40.0	50.95	47.46	46.13 53.96[36]	70.78 (1)	65.29 (4)	-	68.03	70.33 (2)	43.57 (17)	48.71 (2)	54.20
2000–3000	29.68	33.49	30.0	31.05 35.70[36]	38.38 (1)	45.46 (1)	59.25 (1)	47.69	76.24 (6)	60.98 (5)	48.51 (3)	61.91
3000–4000	16.03	17.30	15.07	16.13 18.09[36]	-	-	-	-	60.42 (6)	71.56 (1)	18.46 (2)	50.14
**Average**	**29.44**	**36.50**	**33.92**	**39.23**	**49.96**	**55.37**	**63.72**	**56.72**	**65.93**	**53.66**	**41.81**	**53.80**

The figure in parenthesis represents the number of sample publications reviewed

Asia represents one of the most important centers of knowledge with regard to the use of plant species for treatment of various diseases. Examples are the Ayurveda, Amchi (traditional healing system of Tibet and mountain areas of Nepal), Siddha, Unani, and Chinese systems of medical care [66,67]. Folklore medicinal systems (traditional healing and faith healing) are also important in Nepal. In this context, it is interesting to note that the Himalayan medicinal plants are the major contributors to the aforementioned systems. The topographical characteristics of the Himalayas have resulted in a variety of ecological niches that host diverse medicinal plants [68]. It has been estimated that the Himalayan region harbors over 10,000 species of medicinal and aromatic plants, supporting the livelihoods of about 600 million people living in the area [69]. The Nepal Himalayas include about 2,000 species with medicinal and aromatic values, and more than 1,400 of these are known to be used locally [49-51,53] particularly as medicines [52].

Medicinal and aromatic plant species richness increases along increasing altitude up to 2000 m [70] and then continues to decline. The temperate and alpine zones harbor highly valued medicinal plants [71]. The diversity of medicinal plants in a mid elevation peak model, was comparable to other studies [72-74]. Bhattarai and Vetaas [75] highlighted an insignificant relationship between herbaceous species richness and elevation and climatic factors in the Himalayas. The distribution of medicinal herbs at high altitudes is mainly controlled by both environmental and ecophysiological factors [74,76]. The maximum species richness at 1000–2000 m could be associated with optimum energy and rainfall [75]. The hard boundary effect might also influence the species richness at high elevation [77]. The trans-Himalaya ecosystem is a fragile biome, characterized by a reduced growing season [76], low productivity [78], high intensity of solar radiation, and high degree of resource seasonality [79], resulting in the control of distribution, richness, physiological stress, and metabolic compounds of medicinal plants. The high altitude medicinal plants contain secondary metabolic compounds as an adaptive strategy to reduce the damaging effects [80,81].

### Ethnobotany and ethnomedicine

**Figure 1:**
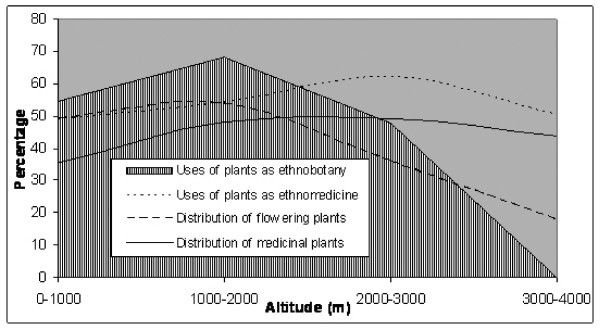
Species richness and usage of plants along altitude in Nepal Himalaya.

Many people use plants as remedies as an alternative or in addition to visiting western health care practitioners. The extent of plant use differs with location. The use of plants for subsistence, medicines, and source for supplementary income is highly variable. About 85% of the rural population of Nepal are said to use herbal remedies [82]. The use of plant resources as herbal remedies is especially important at higher altitudes. We found that the percentage of plants used as medicine increased along increasing altitude but there was no significant trend in increased usage of plants for other purposes. The overall plant use was proportionate to the number of plant species used in ethnomedicine (Fig. 1).

A positive correlation between species richness and number of ethnobotanically useful plants was found, but the relationship was not significant. In contrast, a significant positive correlation was found by Salick et al [83,84]. The ethnobotanical use as percentage of plant species richness (number of enumerated plant species) decreased from east to west Nepal, whereas the ethnomedicinal usage increased from east to west Nepal (Tab. 2). Though there was a record of the lowest number of medicinal plants in west Nepal [36], we found the highest usage of plants as ethnomedicine from the same region. Similar relationships were found by other studies [85,86]. The reason for this could be the excessive utilization of local plant resources as remedies. Lack of modern facilities and services and less development in west Nepal compel the population to use existing plant resources for their immediate needs. Eastern Nepal is comparatively moister than western Nepal [87] and the moisture gradient accounts for differences in plant species richness [70].

High altitude medicinal plants provide quality products [80,81], and this is the reason why they are often the first choice of local users as immediate therapy and by pharmaceutical companies as precious ingredients. Our analysis shows that the proportion of usage of plants as ethnomedicine steadily increased with increasing altitude (Tab. 2). Pohle [88] found, however, an insignificant relationship between the diversity of the flora and the number of wild plants used by local people in mid hills. Higher uses of plants as ethnomedicine at higher altitude (Fig. 1) was attributed to the absence of modern medical facilities [89], and intensive uses of plants as remedies in mountain areas. In remote and high altitude areas, medicinal herbs are the main ingredients of local medicines and the traditional health care system is considered as the main lifeline [90] and frequently the first choice [91]. There is greater dependence among the population upon natural resources [88]. The optimum uses of the limited plant resources revealed a higher percentage of uses in high altitude areas. However, this trend could not be observed in lowland Terai. The usage of plants as ethnomedicine was less in Terai, with the richness of flowering plants and medicinal plant was higher. The low number (48.96%) of ethnomedicinal plants found in Terai was consistent with the findings of Bishokarma et al [[92], (35.15%)] from the same site. Lesser usage of local plants for therapy was attributed to the availability and accessibility of infrastructures, communication, transport, development, and as such less dependant on the availability of plant resources; but a higher preferences towards allopathic medicines. It was also associated with limited knowledge and limited skilled individuals. However, some marginalized communities and tribal groups of lowland Terai, with poor access to the modern medical facilities, were still utilizing local plant resources for local therapy. Subsequently, most communities still use folk herbal remedies as a readily and cheaply available alternative. A decline of the traditional knowledge has been underway for hundreds of years [93], and the knowledge on medicinal plant use is clearly disappearing fast in low lands of Nepal [94].

The higher percentage of ethnomedicinal uses of plants in high altitude areas was due to the preferences given by local people to the traditional herbal remedies, and a situation of having no alternative choices [95,96], as well as poverty and belief in the effectiveness of folklore herbal remedies [46]. The greater usage of plants as herbal remedies in mountain areas is also due to the prevalence of traditional health systems such as folklore medicinal systems and the Amchi system. The traditional healing systems are both culturally acceptable [97,98] and induce locales to use plant resource most. Because of the high level of cohesion and strong cultural links with nature in mountain areas [11,99], local people have a close affinity with locally available resources and easily internalize the resources for their livelihood and needs because these are also readily available.

Home herbal treatment is not merely a medical system but a part of culture [100], and the extensive use of locally available plant resources was observed in mountain areas. Most of the people of mountain areas, particularly elderly people, farmers, and mid-wives, are well acquainted to medicinal herbs and their usage [101], and plants are prescribed to the patients whenever required [102]. The availability of traditional healers is also higher in rural and mountain areas. The use of herbal remedies in mountain areas to alleviate suffering is perhaps as old as the origin of man itself [103], and there is a general trend of increase in the use of medicinal plants [104].

Besides health care and subsistence uses, medicinal plants have a high potential as alternative income-generating sources of the rural hilly populace of the region [105]. Therefore, sustainable use of the resources and conservation of indigenous knowledge of medicinal plants may compliment the income of local people. Medicinal plants are mainly harvested in the wild, traded, and eventually consumed as processed form in lowland cities [106]. Up to 50% of the Nepal's rural household's income is derived from commercial collection of medicinal and aromatic plants [107,108]. The collection of medicinal plants in a sustainable manner is an integrated process with potential for development and conservation [109].

The indigenous knowledge and practice of usage of medicinal plants in rural areas of Nepal is passed down through oral tradition and personal experiences [110]. The knowledge clearly decreased with age. People of ages between 40–60 possessed greater knowledge on identification and uses of medicinal and aromatic plants in Nepal Himalaya [111], which was consistent to the observation of Philips and Gentry [112]. The young generations tend to leave ancestral practices behind, refocusing their interests on treatments offered by western medicine [113]. Due to changing lifestyles, perception as well as social transformation, the plant resource and indigenous knowledge of utilization are being severely degraded [114]. This impact is inevitable to the Nepal Himalayas and plant resources are in great peril. Indigenous knowledge systems are not only of value for the cultures from which they evolve, but also for scientists and planners striving to improve the living conditions in rural societies [115]. Lambert et al [116] pointed out that preserving and enhancing the indigenous plant knowledge and use was equivalent to 'rescuing a global heritage'. Indigenous plant based traditional knowledge and use has become a recognized tool in search for new sources of drugs and pharmaceuticals [117]. In countries like Kenya [118] and Nepal [119], where the indigenous knowledge is predominantly used for utilization of plant resource for various purposes, and high priority needs to be given to the documentation of indigenous knowledge and use of plant resources to help their conservation.

## Conclusion

The richness of medicinal plants was decreasing with increasing altitude, but the percentage of plants used as medicine steadily increased with increasing altitude. This was due to preferences given to herbal remedies in high altitude areas and a situation of having no alternative choices, poverty and belief on effectiveness of folklore herbal remedies. Enhancing the sustainable use and conservation of indigenous knowledge of useful and medicinal plants may benefit and improve the living standard of poor people.

## Competing interests

The authors declare that they have no competing interests.

## Authors' contributions

RMK carried out field research, analyzed the data, and wrote the manuscript and RWB designed the study, supervised the work, and revised the manuscript. Both authors approved the final version of this manuscript.
